# Phenotypic diversity of bread wheat lines with introgressions from the diploid cereal Aegilops speltoides for technological properties of grain and f lour

**DOI:** 10.18699/VJ20.668

**Published:** 2020-11

**Authors:** L.V. Shchukina, I.F. Lapochkina, T.A. Pshenichnikova

**Affiliations:** Institute of Cytology and Genetics of Siberian Branch of the Russian Academy of Sciences, Novosibirsk, Russia; Federal Research Center “Nemchinovka”, Novoivanovskoe, Odintsovsky district, Moscow region, Russia; Institute of Cytology and Genetics of Siberian Branch of the Russian Academy of Sciences, Novosibirsk, Russia

**Keywords:** bread wheat, Ae. speltoides, introgression lines, chromosomal rearrangements, grain vitreousness, protein and gluten content in grain, physical properties of dough, soft grain and endosperm grain hardness, мягкая пшеница, Ae. speltoides, интрогрессированные линии, хромосомные перестройки, стекловидность зерна, содержание белка и клейковины в зерне, физические свойства теста, мягкозерность и твердозерность эндосперма

## Abstract

The creation of varieties adapted to changing environmental conditions, resistant to various pathogens, and satisfying various grain purposes is impossible without using the genetic diversity of wheat. One of the ways to expand the genetic diversity of wheat is to introduce new variants of genes from the genetic pool of congeners and wild relatives into the genotypes of existing varieties. In this study, we used 10 lines from the Arsenal collection created on the genetic basis of the spring variety ‘Rodina’ and the diploid species Aegilops speltoides in the Federal Research Center “Nemchinovka” in 1994. The lines were previously characterized for the presence of translocations and chromosomal rearrangements cytologically and using molecular markers. Technological analyses were performed on grain obtained in Western Siberia and Moscow region. The aim of this study was to establish the possibilities of expanding the phenotypic diversity for technological properties of grain and flour as a result of such hybridization of bread wheat and the diploid cereal Aegilops speltoides. The variety ‘Rodina’ forms a vitreous grain with a high gluten content in Siberia, but has low physical properties of flour and dough. Five derived lines were found to have significantly higher protein and gluten content in grain. The highest values under both growing conditions were found in lines 73/00^i^, 82/00^i^, and 84/00^i^. Two lines (69/00^i^ and 76/00^i^) showed a high flour strength and dough elasticity, characterizing the lines as strong and valuable in quality. These lines can be used for baking bread. Line 82/00^i^ inherited from Ae. speltoides a soft-grain endosperm, which indicates the introgression of the Ha- Sp gene, homoeoallelic to the Ha gene of bread wheat, into ‘Rodina’. Flour of this line is suitable for the manufacture of confectionery without the use of technological additives. The lines generally retained their characteristics in different growing conditions. They can be attracted as donors of new alleles of genes that determine the technological properties of grain and resistance to biotic stresses.

## Abstract (ru)

Создание сортов, адаптированных к изменяющимся условиям окружающей среды, устойчивых к различным патогенам и соответствующих различному целевому назначению зерна, невозможно без ис- пользования генетического разнообразия пшеницы. Одним из путей его расширения является введение в генотипы существующих сортов новых вариантов генов из генетического пула родственных видов и диких сородичей. В настоящем исследовании использованы 10 линий из коллекции «Арсенал», созданных на осно- ве ярового сорта Родина и вида Aegilops speltoides в Научно-исследовательском институте сельского хозяй- ства центральных районов Нечерноземной зоны (ныне Федеральный исследовательский центр «Немчинов- ка») в 1994 г. Линии были ранее охарактеризованы цитологическими и цитогенетическими методами, а также с помощью молекулярных маркеров на наличие замещений и перестроек хромосом. Технологические ана- лизы были выполнены на зерне, полученном в Западной Сибири и Московской области. Цель исследования – установить возможности расширения фенотипического разнообразия пшеницы по технологическим свойствам зерна и муки в результате такой гибридизации. Сорт Родина формирует стекловидное зерно с высоким содержанием клейковины в условиях Сибири, но имеет невысокие физические свойства муки и теста. Обнаружено, что пять производных от него линий имеют достоверно более высокое содержание бел- ка и клейковины в зерне. Наибольшие значения в обоих условиях выращивания установлены у линий 73/00^i^, 82/00^i^ и 84/00^i^. Две линии, 69/00^i^ и 76/00^i^, показали высокую силу муки и упругость теста, характеризующие линии как сильные и ценные по качеству. Зерно этих линий может быть использовано для выпечки хлеба. Линия 82/00^i^ унаследовала от Ae. speltoides мягкозерный эндосперм, что говорит об интрогрессии в геном сорта Родина гена Ha- Sp, гомеоаллельного гену Ha мягкой пшеницы. Мука этой линии пригодна для изготов- ления кондитерских изделий без применения технологических добавок. Линии в основном сохраняли свои особенности в различных условиях выращивания. Они могут быть привлечены в качестве доноров новых аллелей генов, определяющих технологические свойства зерна и устойчивость к биотическим стрессам.

## Introduction

Climate change on the Earth entails a change in growing conditions for crops. Breeders when faced with the new natural challenges, must have a large arsenal of genetic diversity in order to create varieties with the required properties. In Russia, bread wheat is one of the main cereal crops, which grain is used for food, fodder and for technical purposes. Also, grain is an important export item. In 2020, the area under spring bread wheat in Russia was 12.2 million hectares, while the total area under spring crops was 52 million hectares (Ganenko, Belaya, 2020).

For a long time breeding was focused on the obtaining of high-yielding wheat cultivars; this resulted in a loss of valuable and rare alleles that ensure the development of high-quality cultivars with high gluten content in grain. Changes in the spectrum of pathogens and their racial composition also periodically remove many varieties from the use in production. As a result, the gene pool of wheat cultivars becomes narrower in a practical application.

Currently, breeding is faced with the task of obtaining the cultivars that are adapted to changing environmental conditions, resistant to various pathogens and satisfying various end-use grain purposes (baking yeast bread, making pizza, cookies, pancakes, noodles, etc.) (Peña, 2002). This task requires the expansion of the genetic diversity of wheat in many ways. The classic way to solve this problem is to use ancient varieties and genetic collections of wheat in hybridization (Mitrofanova, 2012; Vikram et al., 2016). The alternative way is hybridization of bread wheat with closely related species and wild relatives that carry gene variants that are absent in the genotype of existing cultivars. This pathway is mainly used to search for genes of resistance to biotic and abiotic stresses (Tsitsin, 1958; Vavilov, 1986; Leonova, Budashkina, 2016; Voronov et al., 2019). Introgression of alien genetic material also affects certain grain quality traits (Krupnova, 2013; Shchukina et al., 2017; Alvarez, Guzmán, 2018).

The main grain components of caryopsis that affect the technological properties of grain are gluten (protein) and starch; their composition and content determine the practical use of grain. The search for the genes that can diversify these parameters in wheat during interspecific and intergeneric hybridization is an urgent area of research. Understanding the relationship between the chromosomal rearrangements and introgressions with the formation of the end-use product of grain enlarges the field of work of breeders. The aim of this study was to establish the possibility of enlarging the phenotypic diversity for technological properties of grain and flour in bread wheat due to chromosomal rearrangements resulting from hybridization with diploid cereal Aegilops speltoides.

## Materials and methods

**Genetic material.** We used 10 lines of the spring bread wheat cultivar Rodina from the ‘Arsenal’ collection (69/00^i^, 73/00^i^, 76/00^i^, 77/00^i^, 81/00^i^, 82/00^i^, 84/00^i^, 99/00^i^, 102/00^i^ and 103/00^i^). They were obtained by selecting individual plants with bivalent meiosis from the progeny F_2_M_2_–F_4_M_4_ of asymmetric sex hybrids F1M1 (2n = 49). The asymmetric sex hybrids were obtained from crossing of the cultivar Rodina (2n = 42) with the species Aegilops speltoides (sample k-389 from the VIR collection, St. Petersburg) whose pollen was irradiated with gamma rays at a dose of 10 kR (Lapochkina, 1999). The lines were previously characterized for the presence of substitutions and rearrangements of chromosomes by cytological and cytogenetic methods, as well as using molecular markers.

Establishment of the mechanisms of alien material introgression in wheat genome (the presence of substitutions, translocations) was carried out by studying the nature of chromosome pairing in meiosis in specially obtained F1 hybrids (test line × the original cultivar Rodina). In the lines, the genes for resistance to leaf rust and powdery mildew were identified using microsatellite and STS markers and the test pathotypes of the fungus (Table 1) (Lapochkina et al., 2003, 2005; Gajnullin et al., 2007). The parental cultivar Rodina turned out to be heterogeneous for the chromosomal translocation T1BS/1RS inherited from the cultivar Kavkaz, which was involved in the origin of the cultivar (World Seeds × Kavkaz) (Dorofeev et al., 1987). By analyzing the gliadin storage proteins, the line with the absence of this rearrangement was isolated (hereinafter referred to as cultivar Rodina). This genotype was used as a control in all experiments.

**Table 1.**
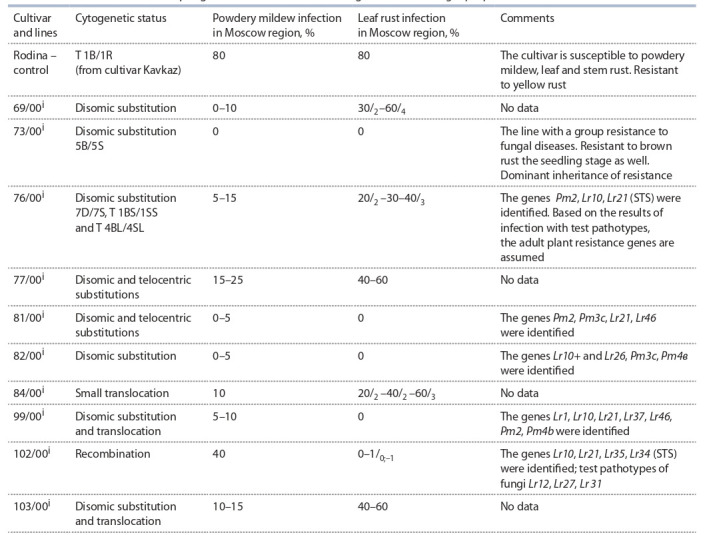
Genetic features of the spring bread wheat lines with introgressions from Aegilops speltoides

**Growing conditions.** The lines and the parent cultivar Rodina were grown with spring sowing in the experimental field of the Institute of Cytology and Genetics of the Siberian Branch of the Russian Academy of Sciences (Akademgorodok, Novosibirsk region) in 2003–2005, 2007, and 2013. On the experimental field of the Federal Research Center “Nemchinovka” (Moscow region), the growing was carried out from 2000 to 2011.

A row seeding scheme was used in tiers with a width of a row 1 m, five rows per sample, 50 grains per row. The soils in Novosibirsk are gray forest, in Nemchinovka – sod-podzolic. Fertilizers were applied to the soil before sowing, in accordance with the agronomic practice adopted for these soils. Harvesting was carried out manually in sheaves, followed by post-harvest ripening for a month, which is necessary for the complete formation of the gluten complex in grain.

According to the agroclimatic zoning of Russia (https://geographyofrussia.com/agroklimaticheskoe-rajonirovanie/), Moscow region and Novosibirsk are situated in the same zone of sufficient moisture for the growing season with droughts in certain years. The meteorological data of the Ogurtsovo station which is the closest in geographical position to the site of the experiments in Novosibirsk, are given in Supplementary Materials 1 and 2^1^. The number of replicates for lines in experimental plots by years in Novosibirsk is indicated in Supplementary Material 3.

Supplementary Materials are available in the online version of the paper: http://www.bionet.nsc.ru/vogis/download/pict-2020-24/appx12.pdf



**Technological analysis** of grain included: determination of thousand grains weight, grain vitreousness, flour particles diameter after grinding, protein and gluten content in grain. We also determined the physical properties of dough, water absorption capacity and mixing characteristics of flour obtained from the grain grown in Novosibirsk.

Thousand grains weight was determined by the express method through weighing of 100 grains. Total grain vitreousness was determined visually after cutting 100 caryopses in half. The indicator of total vitreousness is the sum of fully vitreous and half of the amount of partially vitreous grains (State Standard 10987-76). The average flour particles diameter was determined using a PSH-4 device according the previously described method (Shibayev et al., 1974; Egorov, 2000). Wet gluten content in grain in Novosibirsk was determined by hand washing in water from one gram of meal (State Standard R 54478-2011). The amount of wet gluten was expressed as a percentage of the meal weight. At the Federal Research Center “Nemchinovka” the protein and gluten content in grain was determined on a SpectraStar 2400 Infrared analyzer.

The samples were milled on a laboratory roller mill MLV- 1, with a 70 % flour yield, for further research on alveograph and farinograph devices.

Physical properties of dough were determined on a Chopin alveograph equipped with a fifty-gram kneader (State Standard R 51415-99 with a modification for research work). Flour strength (W, J · 10^–4^), dough elasticity (P, mm) and dough extensibility (L, mm) were determined. Dough balance was calculated as the ratio of elasticity to extensibility (P/L). Water absorption capacity (WAC, %) and mixing characteristics of flour were determined on a Brabender farinograph with a fifty-gram kneader (State Standard ISO-5530-1-2013 with a modification for scientific research). WAC is amount of water (expressed as a percentage) required to form a dough with a consistency of 500 units of farinograph (u. f.). The mixing characteristics included five characteristics: dough formation time (DF, min), dough stability (DS, min), dough liquefaction (DL, u. f.). valorimetric assessment (comprehensive assessment based on the results of the study of flour on a farinograph) (VA, u. val. – valorimeter units). Electrophoresis of endosperm gliadin proteins in the lines was performed as described earlier (Pshenichnikova, Maystrenko, 1995).

**Statistical analysis.** The data for each trait for each genotype were averaged over all years of research (see Suppl. Material 3), and the average deviation was calculated. Student’s t-test was used to determine the significance of differences from the control for each feature. All calculations were performed using Microsoft Office Excel 2013.

## Results

The obtained results are grouped in Tables 2 (milling parameters and wet gluten content in grain), 3 (physical properties of dough) and 4 (mixing characteristics of flour). Thousand grains weight of Rodina was 29.2 g in Novosibirsk and 39.1 g in Nemchinovka (see Table 2). In Novosibirsk its grain was vitreous (80.1 %) and medium-hard (20.4 μm). Wet gluten content in grain reached 36.0 % in Novosibirsk, while in Nemchinovka this value was almost 10 % lower. The flour strength was 145 J · 10^–4^, the elasticity was 56 mm, and the dough extensibility was 108 mm. P/L ratio was low (0.55) (see Table 3). Water absorption capacity of flour in the cultivar was 66.6 %. Dough formation took a little over 3 minutes and it retained the stability for 2 minutes. Dough liquefaction and valorimeter number were 58 u. f. and 59 e. val., respectively (see Table 4). In terms of grain quality, the Rodina variety can be classified as a filler variety (Methods of State Variety Testing…, 1988).

**Table 2.**
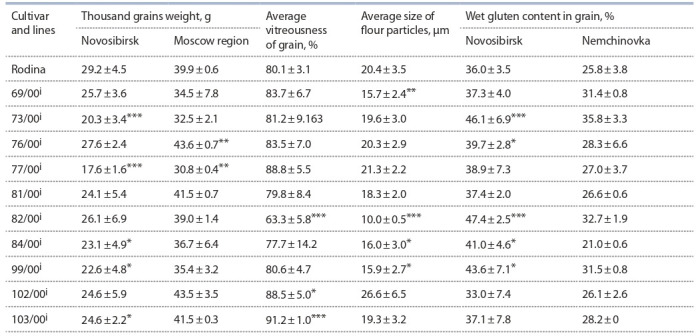
Average long-term indicators of milling parameters and wet gluten content in grain of introgression lines and the cultivar Rodina * р ≤ 0.05; ** р ≤ 0.01; *** р ≤ 0.001.

**Table 3.**
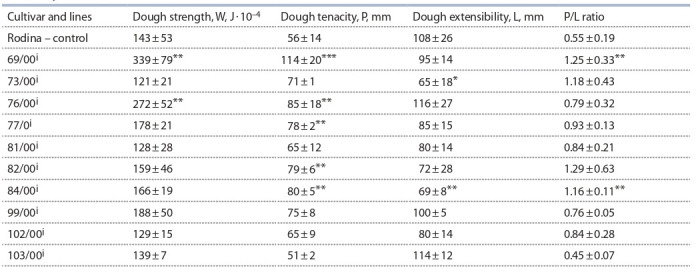
Average values of physical properties of dough in introgression lines and the cultivar Rodina, field, five-year trials (Novosibirsk) р ≤ 0.05; ** р ≤ 0.01; *** р ≤ 0.001.

**Table 4.**
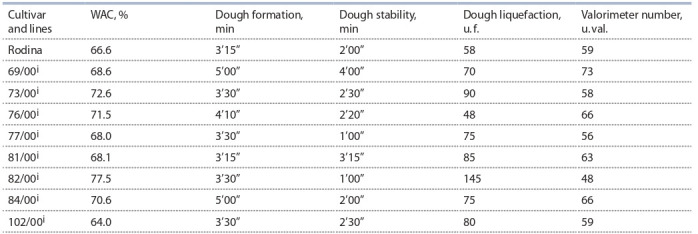
Mixing properties of dough in introgression lines and cultivar Rodina (Novosibirsk, 2013) Note. WAC – water absorbing capacity.

According to Table 2 none of the lines surpassed the control in both geographical areas for thousand grains weight. The trait in Novosibirsk was significantly reduced by the lines 73/00^i^, 77/00^i^, 84/00^i^, 99/00^i^ and 103/00^i^. The smallest grain was in the first two lines, which significantly, by 8.9 and 11.6 g, differed from the control. Of these five lines, three (73/00^i^, 77/00^i^, 99/00^i^) also significantly reduced the trait in Nemchinovka (see Table 2). The correlation between the two regions for this trait was highly significant (r = 0.75, p < 0.001).

Grain vitreousness and flour particles diameter were studied only in Novosibirsk. The lines, in general, did not differ significantly from the Rodina cultivar (see Table 2). The lines 102/00^i^ and 103/00^i^ significantly outperformed the control by 8 and 11 %, respectively. The greatest significant decrease in grain vitreousness was observed in the line 82/00^i^ – 63.3 %, which was accompanied by a twofold decrease in the flour particles diameter (10.0 μm) compared to the original cultivar. The other three lines (69/00^i^, 84/00^i^ and 99/00^i^) also significantly reduced the average diameter of flour particles by about 4 μm, as compared with Rodina (see Table 2).

In the lines 73/00^i^, 76/00^i^, 82/00^i^, 84/00^i^ and 99/00^i^, an increase in gluten content was observed in comparison with Rodina. The highest value was found in the three lines – 73/00i, 82/00i and 99/00i (see Table 2). The same three lines were superior to the parent variety in Nemchinovka. The average gluten content for all the years of research in Novosibirsk was 10 % higher than in Nemchinovka.

Flour strength of the lines 69/00^i^ and 76/00^i^ significantly exceeded the control, by 196 and 129 J · 10^–4^, respectively (see Table 3). Dough elasticity of the introgression lines generally increased. In the lines 76/00^i^, 77/00^i^, 82/00^i^ and 84/00^i^, this trait was significantly higher compared to the control, within the limits of 78–80 mm. Dough elasticity of the line 69/00^i^ has almost doubled. Two lines, 73/00^i^ and 84/00^i^, showed a decreased dough extensibility. P/L ratio of the lines 69/00^i^, 73/00^i^, 82/00^i^ and 84/00^i^ has more than 1.0, that is, it has become more balanced. In the lines 69/00^i^ and 84/00^i^, these changes are significant.

Mixing characteristics of flour in the lines were determined only in one year and in one replication; therefore, it is impossible to draw statistical conclusions about the reliability of the differences between the lines and the control. Nevertheless, some lines are distinguished by a number of parameters (see Table 4). WAC increased in the lines 73/00^i^, 76/00^i^, 82/00^i^ and 84/00^i^. The maximum value of 77.5 % was in the line 82/00^i^ which surpassed the control by more than 10 %. The time of dough formation increased for lines 69/00^i^ and 84/00^i^ compared to the control. Only in the line 69/00^i^ dough stability has doubled. Dough liquefaction mostly increased in the lines. The worst liquefaction value was observed for the line 82/00^i^ – 145 u. f. The valorimetric number in this line was the lowest, only 48 u. val. The line 69/00^i^ had the highest valorimeter number, which outperformed the control by 14 units and consisted 73 e. val. (see Table 4).

## Discussion

Currently, the works are underway to transfer the genes from wild relatives to the genome of bread wheat in order to develop a useful genetic diversity for breeding. Several studies were carried out in relation to the technological properties of grain. In particular, introgression from the wild relative Aegilops markgrafii increased the gluten content in grain and improved other technological parameters (Shchukina et al., 2017). Krupnova (2010) showed the effect of translocations which carry Lr genes from wild relatives Agropiron elongatum, Triticum dicoccum, Agropiron intermedium, T. dicoccoides on the protein content in flour, IDK-1 parameters, sedimentation, falling number and grain test weight.

In our work, we investigated the influence of genetic material from Ae. speltoides, introgressed into the genome of bread wheat cultivar Rodina, on technological characteristics of grain and flour. The transfer of alien genetic material was confirmed by genetic, cytogenetic, and molecular methods (Salina et al., 2001; Adonina et al., 2004, 2012; Gajnullin et al., 2007). Long-term studies have shown that the lines carry genes for resistance to fungal diseases; some of them were identified (see Table 1). Milling properties, gluten content in the grain and dough physical properties were studied in the lines. Variability was found in comparison with the original Rodina cultivar for all technological characteristics studied. Some lines showed a high gluten and protein in grain, variability of milling parameters, variability of rheological and mixing properties of the dough. Ten introgression lines were studied in different years of cultivation and in different geographic regions of Russia. At the same time, it is important to note that some of the discovered new properties were stably preserved under various growing conditions.

**Fig. 1.**
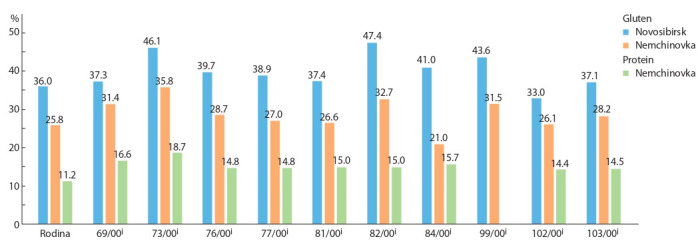
Wet gluten and protein content in grain of introgression lines and the cultivar Rodina grown in Nemchinovka and wet gluten content in grain of the same lines grown in Novosibirsk.

In accordance with Russian and international trade standards gluten content in grain is the most important indicator when determining the wheat grain grade. Figure 1 shows the comparative values of wet gluten content in the grain grown in the Moscow Federal Research Center “Nemchinovka” and in Siberia. The same figure shows the protein content in grain grown in Nemchinovka. The average values in the lines and the parent cultivar grown in Novosibirsk was higher than in Nemchinovka. The difference in gluten content in Rodina was about 13 %, and in the lines, on average, 10 %. As can be seen from the data obtained, the ratio of wet gluten to protein was approximately 2 : 1. Such ratio is typical for bread wheat grain grown under normal cultivation conditions and is consistent with the data obtained by other researchers (Kozmina, 1969; Kulkarni et al., 1987). The line 73/00^i^ was superior for gluten content to all other lines under both growing conditions and showed the highest protein content in grain (see Fig. 1). The lines 76/00^i^, 82/00^i^ and 99/00^i^, in the Moscow region, as well as in Novosibirsk, showed high values of both traits. This suggests that introgressions resulted in the inheritance of the genes that significantly expanded the wheat diversity for protein and gluten content in grain. However, differences were also found in the manifestation of these traits. In the line 69/00i, the protein and gluten content in the Non-Chernozem region was 16.6 and 31.4 %, respectively, which exceeded the control. In Novosibirsk, this line did not differ significantly from Rodina. Significant differences in two growing regions were found for the line 84/00^i^. Under conditions of Novosibirsk it belonged to the group of lines with the highest gluten content, while in the Non-Chernozem region it did not differ from the parent cultivar (see Table 2, Fig. 1).

In the work of Adonina et al. (2012), using fluorescent hybridization with the Spelt1 and pSc119.2 probes in combination with microsatellite markers it was shown that the line 73/00^i^ carries translocations into the short arm of chromosome 1B and the long arm of chromosomes 5B and 6B. Subsequently, these translocations were transferred to separate lines based on the Rodina cultivar (Adonina et al., 2012). The original line 73/00^i^ possesses a group resistance to a spectrum of fungal diseases (see Table 1). But only the obtained line with translocation into 5B chromosome had the maximum resistance to leaf rust (Adonina et al., 2012). Earlier, in this chromosome the loci were found responsible for the high protein and gluten content in grain (Gonzalez-Hernandez et al., 2004; Pshenichnikova et al., 2012). The line 73/00^i^ was distinguished by a decrease in thousand grains weight and accumulation of a high amount of protein and gluten under various conditions. The water absorption capacity of the dough in the line was increased, which is probably due to the high amount of gluten and protein in grain. However, the flour strength was reduced, and dough liquefaction was increased compared to the control which is undesirable when used for food purposes. At the same time, such wheat genotypes can be a valuable source of vegetable protein in the production of feed for livestock and fish farming.

Another line – 82/00^i^, which is characterized by a consistently high protein and gluten content (see Table 2, Fig. 1), simultaneously demonstrated a significant decrease in the vitreousness of the grain and the average diameter of flour particles in comparison with Rodina. The line showed a very high water absorption capacity and a high dough liquefaction. According to the literature data, the Ha locus, located in the subtelomeric region of the short arm of 5D chromosome, is responsible for the hardness and vitreousness of bread wheat (McIntosh et al., 2013). Two closely linked dominant genes in this locus Pina-D1 and Pinb-D1, which encode proteins puroindolines are responsible for the variability of the endosperm structure.

Earlier it was found that the winter line 84/00^w^ from the Arsenal collection with soft-grain endosperm carries the introgression from Ae. speltoides in the form of an entire 5S/5A chromosome substitution. It carries the locus Ha-Sp homeoallelic to the locus Ha (Pshenichnikova et al., 2010). Subsequently, on the basis of the line 84/00^w^, spring supersoftgrain lines were obtained. They combine in their genotypes the homeoallelic loci Ha-Sp of the line 84/98^w^ and Ha the latter being obtained from the soft-grain cultivar Chinese Spring (Simonov et al., 2017). These lines are characterized by a supersoft, mealy endosperm of the caryopsis and by a vitreousness of less than 50 % and flour particle size of 10–12 μm. According to cytogenetic data (see Table 1), the line 82/00^i^ carries a disomic substitution as in the line 84/00^w^. However, the line 82/00^i^ is spring, and it can be assumed that introgression affected only the short arm of chromosome 5A. New spring supersoft grain lines can be obtained using the line 82/00^i^. Flour of such lines is suitable for the manufacture of confectionery products without the use of technological additives (Peña, 2002).

The same introgression in the proposed region could lead to an increase in wet gluten content in grain. Earlier, in the Weimai × Yannong hybrid wheat population, in the region of chromosome 5A marked with the molecular markers Xcfa2163.2-Xcwm216 the main locus QGpc.WY-5A.1 responsible for 53 % of the phenotypic variability for protein accumulation in grain, and the locus QWgc.WY-5A.2 responsible for 36 % of the phenotypic variability for wet gluten content were co-localized (Li et al., 2012). It should also be noted that the line 82/00^i^, in contrast to the line 73/00^i^, had thousand grains weight comparable to the original variety (see Table 2). This indicates the possibility of selection for high gluten and protein content without loss of yield. The line was characterized by a high resistance to fungal diseases carrying the identified resistance genes Lr10 and Lr26, Pm3c, Pm4b (see Table 1). This level of resistance possibly is also provided by introgression.

**Fig. 2.**
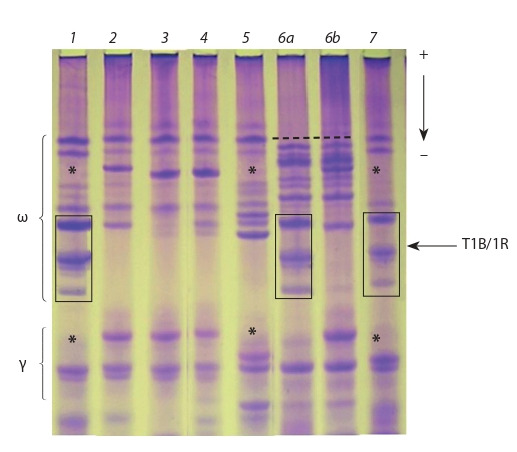
Electrophoregram of gliadin components in the introgression lines. The asterisks indicate the components of γ- and ω-gliadin that are absent as a result of translocation. The dashed line indicates the rearrangement in locus Gli-D1. 1 – 84/00^i^; 2 – 82/00^i^; 3 – 76/00^i^; 4 – cultivar Rodina; 5 – 73/00^i^; 6a, 6b – 69/00^i^; 7 – 81/00^i^.

The original cultivar Rodina was characterized by low rheological and mixing properties. The cultivar Kavkaz – the carrier of the 1BS/1RS rye translocation is present in its pedigree. The cultivar was heterogeneous for this trait. This translocation is known to significantly impair dough physical properties (Martin, Stewart, 1990) since it affects the composition of high molecular weight glutenins and gliadins. These gluten proteins determine the balance between the elasticity and extensibility of dough. By analyzing the component composition of gliadins (Fig. 2), we selected the line of Rodina which does not contain the translocation. Nevertheless, the physical properties of this line remained low (see Tables 2, 3). According to electrophoretic data, the 1BS/1RS translocation was inherited by the lines 81/00^i^, 84/00^i^ and 69/00^i^ (see Fig. 2). The last line was heterogeneous for this trait. Basically, these lines were also characterized by a low flour strength like Rodina. Only one line, 69/00^i^, showed the average value of flour strength allowing to classify the line as strong in quality and use it as an improver for baking purposes. The line 69/00^i^ is characterized by the absence of the most slowly moving components of the ω-fraction of gliadins (see Fig. 2) which are controlled by the Gli-D1 locus of chromosome 1DS (Pshenichnikova, Maystrenko, 1995). The locus is closely linked to the Glu-D1 locus, coding high-molecular subunits of glutenins, which largely determine the strength of flour and elasticity. Probably, this region of the chromosome has undergone a recombination as a result of distant crossing. Interestingly, the presence of the 1BS/1RS translocation does not impair the physical properties of this line.

Disomic substitutions were found in the lines 76/00^i^; and 81/00^i^; by cytological methods. Molecular methods identified the complete substitution of 7D chromosome for 7S chromosome from Ae. speltoides (Adonina et al., 2004). The line 81/00^i^; additionally carries the 1ВS/1RS translocation, and the line 76/00^i^; carries a translocation into the short arm of chromosome 3A. The line 76/00^i^; differs from the line 81/00^i^; in a number of technological parameters for the better. It can be classified as valuable in quality and used as an improver. Gluten content in the line 76/00^i^; was significantly increased; gluten was of better quality. Flour strength in the line reached 272 J · 10^–4^, and the dough became more elastic (see Table 3). Mixing flour parameters and water absorption capacity have improved (see Table 4). It can be assumed that the substitution of chromosome 7D for 7S has a positive effect on the quality parameters of flour if the rye secalins are absent in gluten composition. In addition, it was shown that the line 76/00^i^; carries the 1BS/1SS translocation (see Table 1). Earlier, it has already been noted that the introgression into the short arms of the first homoeologous group chromosomes from species of the genus Aegilops improves baking properties (Alvarez, Guzmán, 2018).

## Conclusion

Ten spring lines from the ‘Arsenal’ collection, selected initially for the resistance to powdery mildew or leaf rust, were for the first time studied for a wide range of technological parameters, including milling parameters, gluten and protein content in grain and physical properties of flour and dough. Our research has shown that introgressions from the species Ae. speltoides significantly expand the genetic diversity of common wheat for these properties and, as a consequence, the possibilities of the final use of grain and flour. In this work, the lines were identified that combine the new variability for various technological traits with the resistance to various fungal diseases. The lines generally retained their characteristics under different growing conditions in different growing years. They can be involved in breeding work as donors of a complex of agronomically valuable traits.

## Conflict of interest

The authors declare no conflict of interest.
